# Examining Optimism, Psychosocial Risks, and Cardiovascular Health Using Life's Simple 7 Metrics in the Multi-Ethnic Study of Atherosclerosis and the Jackson Heart Study

**DOI:** 10.3389/fcvm.2021.788194

**Published:** 2021-12-15

**Authors:** Jee Won Park, Akilah J. Dulin, Belinda L. Needham, Mario Sims, Eric B. Loucks, Joseph L. Fava, Laura A. Dionne, Matthew M. Scarpaci, Charles B. Eaton, Chanelle J. Howe

**Affiliations:** ^1^Center for Epidemiologic Research, Brown University, Providence, RI, United States; ^2^Department of Epidemiology, Brown University, Providence, RI, United States; ^3^Department of Behavioral and Social Sciences, Center for Health Promotion and Health Equity Research, Brown University, Providence, RI, United States; ^4^Department of Epidemiology, University of Michigan, Ann Arbor, MI, United States; ^5^Department of Medicine, University of Mississippi Medical Center, Jackson, MS, United States; ^6^Centers for Behavioral and Preventive Medicine, The Miriam Hospital, Providence, RI, United States; ^7^Hassenfeld Child Health Innovation Institute, Brown University, Providence, RI, United States; ^8^Department of Family Medicine, Warren Alpert Medical School of Brown University, Providence, RI, United States

**Keywords:** optimism, resilience, psychosocial factors, effect measure modification, cardiovascular health (CVH)

## Abstract

**Background:** Optimism has been shown to be positively associated with better cardiovascular health (CVH). However, there is a dearth of prospective studies showing the benefits of optimism on CVH, especially in the presence of adversities, i.e., psychosocial risks. This study examines the prospective relationship between optimism and CVH outcomes based on the Life's Simple 7 (LS7) metrics and whether multilevel psychosocial risks modify the aforementioned relationship.

**Methods:** We examined self-reported optimism and CVH using harmonized data from two U.S. cohorts: Multi-Ethnic Study of Atherosclerosis (MESA) and Jackson Heart Study (JHS). Modified Poisson regression models were used to estimate the relationship between optimism and CVH using LS7 among MESA participants (*N* = 3,520) and to examine the relationship of interest based on four biological LS7 metrics (body mass index, blood pressure, cholesterol, and blood glucose) among JHS and MESA participants (*N* = 5,541). For all CVH outcomes, we assessed for effect measure modification by psychosocial risk.

**Results:** Among MESA participants, the adjusted risk ratio (aRR) for ideal or intermediate CVH using LS7 comparing participants who reported high or medium optimism to those with the lowest level of optimism was 1.10 [95% Confidence Interval (CI): 1.04–1.16] and 1.05 (95% CI: 0.99–1.11), respectively. Among MESA and JHS participants, the corresponding aRRs for having all ideal or intermediate (vs. no poor) metrics based on the four biological LS7 metrics were 1.05 (0.98–1.12) and 1.04 (0.97–1.11), respectively. The corresponding aRRs for having lower cardiovascular risk (0–1 poor metrics) based on the four biological LS7 metrics were 1.01 (0.98–1.03) and 1.01 (0.98–1.03), respectively. There was some evidence of effect modification by neighborhood deprivation for the LS7 outcome and by chronic stress for the ideal or intermediate (no poor) metrics outcome based on the four biological LS7 metrics.

**Conclusion:** Our findings suggest that greater optimism is positively associated with better CVH based on certain LS7 outcomes among a racially/ethnically diverse study population. This relationship may be effect measure modified by specific psychosocial risks. Optimism shows further promise as a potential area for intervention on CVH. However, additional prospective and intervention studies are needed.

## Introduction

Cardiovascular disease (CVD) is the leading cause of death in men and women, and in most racial/ethnic groups in the United States (U.S.) (1). In 2018, the prevalence of CVD in the U.S. was highest among White non-Hispanic adults (2). However, the mortality rate among Black non-Hispanic adults was higher than White non-Hispanic adults and was almost 2-fold higher than Hispanic adults (3). These racial/ethnic health disparities are shaped by structural racism that negatively impacts the social determinants of health (4), resulting in greater exposures to psychosocial risks and engagement in adverse health behaviors related to CVD (5–10).

To improve an individual's cardiovascular health (CVH) and reduce and prevent CVD incidence and mortality, the American Heart Association proposed Life's Simple 7 (LS7) (11). CVH is determined by LS7 metrics that consist of three behavioral (smoking, physical activity, diet) and four biological factors (body mass index (BMI), blood pressure (BP), total cholesterol, and fasting glucose) (11). This measure, which can be categorized into ideal, intermediate, or poor CVH for each indicator or the sum total of LS7 metrics, has been examined in various studies and shown to be a strong independent marker for CVD outcomes (12–14). Specifically, there is growing evidence based on several longitudinal and meta-analytic studies of CVH that show the relationship between higher CVH scores and lower risk of CVD and mortality (12, 15–20).

Psychosocial risks may also affect CVH and contribute to observed racial/ethnic disparities (6, 7). Psychosocial risk has been defined as social or psychological factors that negatively influence health (21). Prior work suggests that psychosocial risks at multiple levels, such as experiencing chronic stress (individual-level), discrimination (interpersonal-level), and low neighborhood socioeconomic position (SEP), are associated with poor CVH outcomes (5–7), disproportionately impact racial/ethnic minorities like African American or Black adults, and are important to address in order to improve CVH (22). However, compared to psychosocial risks, resilience may be protective and a more readily malleable intervention target for improving CVH and reducing related racial/ethnic disparities (23–29).

Resilience is defined as an individual's ability to overcome and positively adapt to adversities to reduce the harmful effects of adverse situations on health and development (30, 31). Individuals can utilize resilience resources at multiple levels, for example, at the individual, interpersonal, or neighborhood level (32). Furthermore, conceptual frameworks such as the Reserve Capacity Model posit that resilience resources help individuals adapt to the adverse effects of threats to CVH (33, 34). This hypothesis is supported by a recent systematic review and meta-analysis (35). In particular, greater individual-level resilience resources are associated with lower adverse CVD outcomes. These encouraging results may be due to individual-level resilience resources operating through indirect pathways that may increase healthy behaviors and reduce adverse CVD outcomes when individuals are exposed to psychosocial risks (28).

Optimism, which can be defined as an individual's positive mindset with beliefs or expectations that good things will happen, is a potentially modifiable individual-level resilience resource that may affect CVH (36, 37). Optimism may positively impact CVH through biobehavioral mechanisms; for example, an optimistic individual may use more adaptive coping strategies to overcome adversities and have reduced inflammation and improved blood pressure to enhance cardiovascular functioning (22, 38–40). A limited number of cross-sectional studies have demonstrated a positive relationship between optimism and ideal CVH. For instance, cross-sectional data from the Multi-Ethnic Study of Atherosclerosis (MESA) showed that participants in the highest quartile of optimism were more likely to have ideal CVH when compared to the least optimistic group. In another cross-sectional study in the Hispanic Community Health Study/Study of Latinos (HCHS/SOL), higher individual optimism levels were associated with higher CVH scores (38). In one longitudinal study among Black and White adults in the U.S., optimism was positively associated with better CVH over time (41). Thus, individual-level resilience resources, such as optimism, have the potential to be an effective intervention target in improving CVH (37, 40–42).

Currently, there are several research gaps in the literature on optimism and CVH. First, the link between optimism and individual components of LS7, such as smoking or physical activity, has been documented (26, 41, 42). However, there is a lack of studies that examine the composite LS7 measure prospectively. Second, most studies on optimism have not been conducted in populations that include racial/ethnic minorities despite some racial/ethnic minorities experiencing a considerable burden of CVD (1–3). Third, psychosocial risks, such as stress and discrimination associated with poor CVH (5–7), may differ across populations. For example, Black non-Hispanic adults and people in low socioeconomic positions (SEP) experience more psychosocial risks compared to other groups (43–45). These differences in psychosocial risks may result in differences in the benefits of resilience resources across populations. However, most optimism research in the context of CVH has not assessed for effect measure modification by psychosocial risks, one at a time, at the individual, interpersonal, or neighborhood level.

Given the previously mentioned gaps in the literature, the objective of this study is to examine the relationship between optimism and ideal CVH prospectively, where composite LS7 metrics will measure CVH outcomes. In addition, we will assess if multilevel psychosocial risks modify this relationship.

## Methods

### Study Population

Data in this study come from a retrospective data harmonization that integrated longitudinal data from three U.S. cohorts on CVD: the Jackson Heart Study (JHS), MESA, and the Mediators of Atherosclerosis in South Asians Living in America (MASALA) study. These cohorts included measures of resilience, psychosocial risks, and neighborhood-level socioeconomic factors in Exams 1, 2, and/or their annual follow-up interviews. Also, the three cohorts assessed most, if not all, LS7 metrics during all study exams and included measures on incident CVD events during the exams or the annual follow-up interviews. Additional details on these three cohorts can be found elsewhere (46–48).

JHS is a prospective cohort study among African American men and women in the Jackson, Mississippi metropolitan area who were 21 years or older. Participant data from three JHS exams (Exam 1: September 2000-March 2004, Exam 2: October 2005-December 2008, Exam 3: February 2009-January 2013) and the annual follow-up interviews were included in this study. The annual follow-up interviews in JHS were conducted approximately every 12 months from the date of the first examination (i.e., Exam 1) to collect information on clinical events and additional health-related measures, such as optimism and neighborhood social cohesion (49). MESA is a prospective cohort study of White non-Hispanic, African American, Asian, and Hispanic men and women from six U.S. sites (New York, New York, Baltimore, Maryland, Chicago, Illinois, Los Angeles, California, Minneapolis-St. Paul, Minnesota, and Winston-Salem, North Carolina) who were over 45 and free of clinical CVD at Exam 1. MESA participant data from Exams 1-5 were included in this study (Exam 1: July 2000-August 2002, Exam 2: September 2002-February 2004, Exam 3: March 2004-September 2005, Exam 4: September 2005-May 2007, Exam 5: April 2010-December 2011). MASALA study participants were excluded from the harmonized study population because MASALA did not assess for optimism.

The three cohort studies (JHS, MASALA, and MESA) were approved by the Institutional Review Board (IRB) at the participating institutions, and the secondary analysis of the data analyzed in this paper was approved by the IRB at Brown University (Providence, Rhode Island).

### Measures

The exposure variable was time-fixed, self-reported optimism measured during the second annual follow-up telephone interview (between Exams 1 and 2) in JHS and Exam 2 in MESA. Because we did not have the exact date of the second annual follow-up interview in JHS when optimism was assessed, we assumed that optimism was assessed 2 years after Exam 1 for all JHS participants included in our study. Optimism was measured by a 6-item questionnaire, Life Orientation Test-Revised (LOT-R) (50). The LOT-R demonstrated an acceptable level of reliability with a Cronbach's alpha of 0.69 in the JHS and MESA data combined (0.64 in the JHS alone and 0.73 in MESA alone). Since the optimism scale does not have a clinical cut-off value and to be consistent with prior research on optimism, we considered optimism as tertiles, i.e., categorized into low, medium, and high (40).

Potential confounding variables included time-fixed, individual-level (religiosity), interpersonal-level (social support), or neighborhood-level (social cohesion) resilience resources. A 6-item Daily Spiritual Experiences Scale (51) measured religiosity during Exam 1 in JHS and 2 in MESA. Social support was measured by summing the items that captured the constructs of “someone to talk to,” “someone to give advice,” “someone to be there emotionally,” and “someone to help with chores” from the Interpersonal Social Support Evaluation List (52) in JHS and Social Support Inventory (53) in MESA, both during Exam 1. Neighborhood social cohesion was based on a 5-item perceived social environment/cohesion scale (54) during the third annual follow-up interview in JHS (between Exams 1 and 2) and Exam 1 in MESA. All measures were self-reported and dichotomized into a binary variable using a median split (high vs. not high) consistent with previous studies (40, 55, 56).

Other confounding variables included age (continuous), sex (male, female), race/ethnicity (White non-Hispanic, Asian, African American, Hispanic), geographical region (West, South, Midwest, Northeast), nativity (U.S.-born, other), marital status (married, never married/separated/divorced/widowed), self-rated health (good, not good), health insurance type (public/private, none), self-history of CVD and stroke (yes, no), and family history of CVD and stroke (yes, no). These variables were self-reported, time-fixed, and assessed during Exam 1 in both cohorts. Further, based on previous literature (42), we considered all confounding variables to be potentially related to the exclusion of some participants with missing data or censoring. Thus, all confounding variables were used to minimize selection bias.

Psychosocial risks that negatively influence physical health at the individual level (anger, depressive symptoms, chronic stress, education, employment status, income), interpersonal level (discrimination), and neighborhood level (neighborhood deprivation, neighborhood safety) were considered as potential effect measure modifiers. These psychosocial factors may alter the optimism-CVH relationship based on previous literature (5–7, 42). Anger, measured using the Anger-Out in JHS and State-Trait in MESA from the Spielberger State-Trait Anger Expression Inventory (57), was considered as tertiles (low, medium, high). Depressive symptoms were measured using the Center for Epidemiologic Studies-Depression (CES-D) scale (58), and a cut-off of 16 was used to determine a binary depression variable. Chronic stress, measured by summing items related to stress from medical, job, finances, and relationships from the Global Perceived Stress Scale developed for JHS and the Chronic Burden scale for MESA (59), was categorized into tertiles. Education was categorized into less than high school, high school or some college, and college degree or more. Annual family income was categorized into $0-$19,999, $20,000-$49,999, and more than $50,000. Employment status was dichotomized into employed at least part-time and unemployed. Perceived discrimination, which was measured by the Everyday Discrimination Scale (60), was categorized into tertiles. Neighborhood deprivation was measured by a census-tract level neighborhood summary score of socioeconomic indicators using principal component factor analysis (61), and was categorized into tertiles. Briefly, a summary score indicating the neighborhood socioeconomic deprivation was created by combining the z-scores of six measures derived from the 2000 U.S. Census and American Community Survey 2005–2009 and 2007–2013. These measures included median household income, median housing value, percentage of households with interest, dividend, or net rental income, percentage of adults age 25 or older with a high school degree or higher, percentage of adults age 25 or older with a Bachelor's degree or higher, and percentage of people employed in executive, managerial, or professional occupation. Higher neighborhood summary scores indicated lower neighborhood deprivation (i.e., a better socioeconomic context) (61). Neighborhood safety was a 1-item neighborhood safety from crime question and was dichotomized into safe and not safe. All potential effect measure modifiers except for neighborhood deprivation were self-reported, assessed during Exam 1 in both cohorts, and the categorization of each measure was consistent with past studies in JHS and MESA (62–66). Further, all potential effect measure modifiers were considered to be potential sources of confounding or selection bias.

The primary outcome variable was CVH determined by non-repeated-measures LS7 metrics at MESA Exam 5. The poor categories for each of the LS7 metrics were as follows: (1) poor smoking: current smokers; (2) poor BMI: ≥ 30 kg/m^2^; (3) poor physical activity: 0 min per week; (4) poor diet: 0-1 healthy dietary components; (5) poor cholesterol: ≥ 240 mg/dL; (6) poor BP: ≥140/90 mm Hg; (7) poor blood glucose: ≥ 126 mg/dL. Additional details on each category of CVH (poor, intermediate, and ideal) can be found elsewhere (11, 67, 68). These metrics were based on self/proxy-reported and/or physical examinations at Exam 5. Scores of 0, 1, 2 for each poor, intermediate, and ideal metric, respectively, were summed to represent a total CVH score ranging from 0 to 14. CVH was considered as a binary indicator comparing ideal or intermediate CVH (scores ranging between 8 and 14) with poor CVH (score ranging between 0 and 7) due to a small number of study participants with ideal CVH. JHS study participants were excluded from analyses that used the primary outcome variable because there was no complete LS7 CVH metric (ranging from 0 to 14) available after optimism assessment for these participants. Thus, we only included MESA participants at Exam 5 in analyses that used the primary outcome variable.

The secondary outcome variables used repeated-measures of the four biological LS7 metrics (BMI, BP, blood glucose, and cholesterol) assessed during exams subsequent to when optimism was assessed, i.e., JHS Exams 2-3 and MESA Exams 3-5. Because some LS7 metrics were not collected during follow-up exams, we created the secondary outcome variables by adapting methods used in previous studies (69, 70). We categorized the variables into “all ideal metrics,” “at least 1 intermediate but no poor metrics,” and “at least 1 poor metric.” Due to a small sample size in the “all ideal” category, we created a binary outcome variable to compare “ideal or intermediate (no poor) metrics” vs. “at least 1 poor metric.” Additionally, we compared participants with 0-1 poor metrics (lower cardiovascular risk) with 2-4 poor metrics (non-lower cardiovascular risk).

### Statistical Analysis

Characteristics at the relevant interview or relevant exams were compared between the included and a subset of the excluded participants (i.e., participants without an outcome measurement), using chi-squared and Wilcoxon Mann-Whitney tests.

Primary analyses from a sample of the MESA participants were conducted using unadjusted and adjusted modified Poisson regression models while accounting for within neighborhood clustering (71). Modified Poisson models allow for the estimation of risk ratios from correlated binary outcomes in prospective data, and it also provides valid estimates and standard errors for clustered data (71). To estimate the overall relationship between optimism and ideal CVH in the MESA study, time-fixed optimism was included in an unadjusted model. The corresponding adjusted model included time-fixed optimism and covariates to minimize sources of confounding and selection bias. Restricted quadratic splines (RQS) with 4 knots at unequal intervals (i.e., 5, 35, 65, and 95th percentiles) were used to model the continuous age variable in all adjusted models to facilitate correct model specification and in turn, better control for potential sources of confounding and selection bias (72). To assess effect measure modification by each psychosocial risk one at a time, each adjusted model was revised as needed to include product terms between optimism and the relevant psychosocial risk. A global chi-squared test was conducted to obtain a *p*-value that indicated whether at least one of the product term coefficients between optimism and the relevant psychosocial risk measure was different from zero.

Secondary analyses from a harmonized sample of JHS and MESA participants were conducted using unadjusted and adjusted repeated-measures modified Poisson regression models with observations clustered at the neighborhood level (71). To estimate the overall relationship between optimism and the secondary outcomes, the unadjusted model included time-fixed optimism, time-updated visit, and a product term between optimism and visit (optimism-visit). The corresponding adjusted model additionally included covariates to control for confounding and selection bias. For the continuous age variable, RQS with 4 knots at unequal intervals were also included. Since neighborhood social cohesion was measured after optimism was assessed in JHS, we did not adjust for neighborhood social cohesion in the secondary analyses. For parsimony, we also analyzed the unadjusted and adjusted models without the optimism-visit product term. Similar to the primary analyses, we assessed for effect measure modification by psychosocial risk one at a time with and without the optimism-visit product term. In the adjusted models for effect measure modification that included the optimism-visit product term, we also had a 3-way product term between optimism, visit, and the relevant psychosocial risk, as well as all relevant lower-order product terms (73). Again, a global chi-squared test was conducted to obtain a *p*-value for relevant product terms.

The harmonized data that included JHS and MESA studies had differing times between subsequent exams. With no exact exam dates available in the MESA study, we assumed the 1st day of the month for the exam date. By design, JHS conducted exams every 4–5 years, and MESA conducted exams every 2–3 years. We, therefore, grouped the follow-up times into two 4-year bins (i.e., visits 1 and 2) given the maximum of 8 years of follow-up time. This 4-year binning approach resulted in some participants having more than one exam within a 4-year interval. Therefore, we took the furthest follow-up observation as our visit so that all participants had one observation per bin. We censored participants at the minimum of the first missed visit due to missing data because of death or another reason and reaching the administrative censoring visit (i.e., visit 2). Because of the small proportion of deaths during the follow-up period (7.0%), we treated death as a censoring event rather than an event that results in the CVH outcome being undefined (74, 75).

Based on the published literature (76) that indicates that valid inference can be made using an independent working correlation structure when analyzing clustered outcome data with generalized estimating equations (GEE), we selected an independent working correlation structure for both the primary and secondary analyses to account for within-neighborhood clustering. For the secondary analyses, we assumed that subjects were nested within the neighborhood. Therefore, clustering on the neighborhood in the secondary analyses should also account for clustering by subject (71).

For sensitivity analyses, we further adjusted for years of follow-up since the exposure (i.e., optimism) assessment in our primary analyses by using RQS with 4 knots at unequal intervals. In our secondary analyses, we repeated all analyses by clustering on the subject, and not the neighborhood, to see if inferences changed. We also repeated all primary and secondary analyses assuming an exchangeable working correlation structure. Last, we conducted the secondary analyses stratified by cohort.

We used an alpha level of 0.05 for statistical tests and calculated 95% CIs for all relevant analyses. Findings (e.g., point estimates, confidence intervals, and *p*-values) were interpreted based on data compatibility rather than solely on statistical significance (i.e., *p* ≤ 0.05) to reflect the recent literature on significance and hypothesis testing (77–79). Therefore, the inclusion of the null value in the confidence interval or a *p* > 0.05 was by itself not deemed as sufficient evidence for the absence of an association or effect measure modification (78, 80). All analyses were performed using SAS 9.4 (SAS Institute, Inc., Cary, North Carolina).

## Results

### Primary Analyses

**Figure 1:**
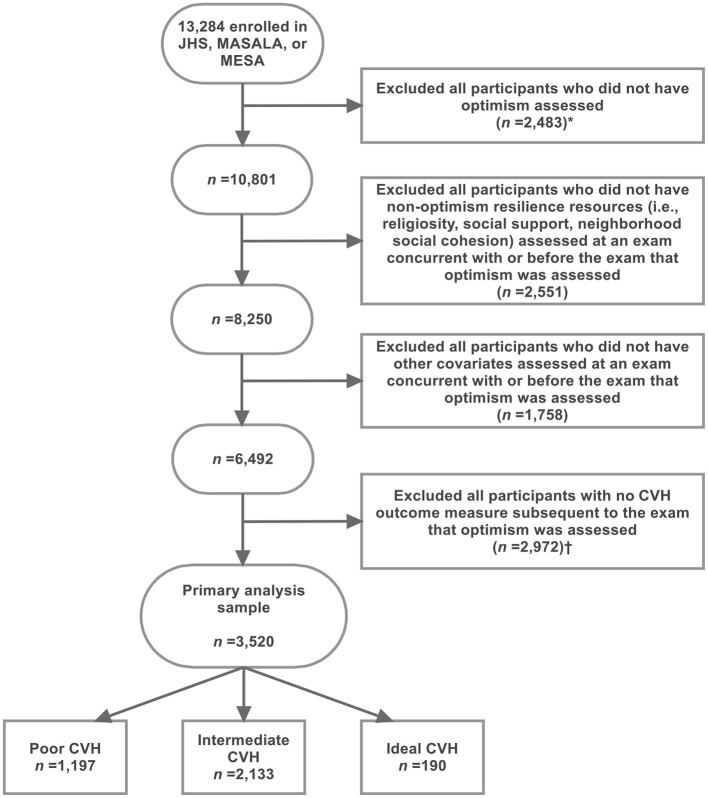
Exclusion criteria applied to identify 3,520 MESA participants who were included in the primary analysis sample. *This exclusion step resulted in the exclusion of all MASALA participants because optimism was not assessed in MASALA. This exclusion step resulted in the exclusion of all remaining JHS participants because Life's Simple 7 (LS7) metrics for CVH outcome was not assessed subsequent to the assessment of optimism in JHS. CVH, cardiovascular health; JHS, Jackson Heart Study; MASALA, Mediators of Atherosclerosis Among South Asians Living in America; MESA, Multi-Ethnic Study of Atherosclerosis.

A total of 3,520 MESA participants were included in the primary analyses (Figure 1). Table 1 shows the demographic characteristics and the distribution of the tertiles of optimism, psychological risk, and resilience measures of the included and a subset of the excluded participants at relevant exams. The median (25–75th percentile) age and follow-up time since the exposure assessment of the included participants were 60 (52–67) and 7.8 (7.6–8.0) years, respectively. Most included participants were female (54.6%), White non-Hispanic (41.8%), U.S.-born (70.9%), and married (64.0%). Most had good self-rated health (92.4%), public or private health insurance (92.6%), a family history of CVD (58.2%), and a high school or some college education (46.5%). Further, most were employed at least part-time (55.1%), reported more than $50,000 annual family income (45.0%), were not depressed (88.2%), and reported that their neighborhood was safe (84.2%). Ideal, intermediate, and poor CVH assessed at Exam 5 was reported in 5.4, 60.6, and 34.0% of the included participants, respectively. In comparison to the excluded participants, the included participants were similar in age and more likely to be male, White non-Hispanic, non-U.S.-born, married, have good self-rated health, have health insurance, report a family history of CVD, achieve higher levels of education, have a higher annual family income, and were less likely to be depressed.

**Table 1:** Characteristics at the exam that optimism was assessed or exams concurrent with or before optimism assessment comparing the included and a subset of the excluded MESA participants (i.e., participants with no CVH outcome measure subsequent to the exam that optimism was assessed) in the primary analyses.

**Characteristic**	**Included**		**Excluded**		*P*-value^*^
	**(*****n*** **=** **3,520)**		**(*****n*** **=** **2,972)**		
	***N***	**%**	***N***	**%**	
**Optimism**^†^ **at Exam 2**					
High	1,045	29.7	924	31.1	0.03
Medium	1,130	32.1	1,006	33.9	
Low	1,345	38.2	1,042	35.1	
**Follow-up years** ^‡^	7.8 (7.6–8.0)				
**Age**^‡^ **in years at Exam 1**	60 (52–67)		59 (49–69)		<0.01
**Sex at Exam 1**					
Female	1,922	54.6	1,723	58.0	<0.01
Male	1,598	45.4	1,249	42.0	
**Self-reported race/ethnicity at Exam 1**					
White non-Hispanic	1,471	41.8	430	14.5	<0.01
Asian	366	10.4	152	5.1	
African American	899	25.5	2,056	69.2	
Hispanic	784	22.3	334	11.2	
**Nativity at Exam 1**					
Other	1,024	29.1	432	14.5	<0.01
U.S.-born	2,496	70.9	2,540	85.5	
**Region at Exam 1**					
West	571	16.2	366	12.3	<0.01
South	624	17.7	1,870	62.9	
Midwest	1,273	36.2	340	11.4	
Northeast	1,052	29.9	396	13.3	
**Marital status at Exam 1**					
Never married, separated/divorced, widowed	1,267	36.0	1,246	41.9	<0.01
Married	2,253	64.0	1,726	58.1	
**Self-rated health**^§^ **at Exam 1**					
Not good	266	7.6	553	18.6	<0.01
Good	3,254	92.4	2,419	81.4	
**Health insurance at Exam 1**					
None	261	7.4	357	12.0	<0.01
Public or Private	3,259	92.6	2,615	88.0	
**Family history of CVD and stroke at Exam 1**					
No	1,473	41.9	1,310	44.1	0.07
Yes	2,047	58.2	1,662	55.9	
**Education at Exam 1**					
Less than high school	476	13.5	482	16.2	<0.01
High school or some college	1,637	46.5	1,398	47.0	
College degree or more	1,407	40.0	1,092	36.7	
**Employment at Exam 1**					
Unemployed	1,581	44.9	1,499	50.4	<0.01
Employed (Part/full-time)	1,939	55.1	1,473	49.6	
**Income at Exam 1**					
$0–$19,999	638	18.1	801	27.0	<0.01
$20,000–$49,999	1,299	36.9	1,086	36.5	
$50,000+	1,583	45.0	1,085	36.5	
**Anger**^†^ **at Exam 1**					
Low	1,370	38.9	1,087	36.6	<0.01
Medium	1,206	34.3	848	28.5	
High	944	26.8	1,037	34.9	
**Depression (CES-D≥16) at Exam 1**					
No	3,103	88.2	2,432	81.8	<0.01
Yes	417	11.9	540	18.2	
**Chronic stress**^†^ **at Exam 1**					
Low	1,729	49.1	917	30.9	<0.01
Medium	861	24.5	703	23.7	
High	930	26.4	1,352	45.5	
**Discrimination**^†^ **at Exam 1**					
Low	1,026	29.2	828	27.9	<0.01
Medium	1,318	37.4	933	31.4	
High	1,176	33.4	1,211	40.8	
**Neighborhood deprivation**^†^ **at Exam 1**					
Low	1,242	35.3	686	23.1	<0.01
Medium	1,193	33.9	755	25.4	
High	1,085	30.8	1,531	51.5	
**Neighborhood safety at Exam 1**					
Safe	2,965	84.2	2,111	71.0	<0.01
Not safe	555	15.8	861	29.0	
**Religiosity at Exam 2**					
Not high	1,675	47.6	1,035	34.8	<0.01
High	1,845	52.4	1,937	65.2	
**Social support at Exam 1**					
Not high	1,355	38.5	906	30.5	<0.01
High	2,165	61.5	2,066	69.5	
**Neighborhood social cohesion at Exam 1**					
Not high	1,632	46.4	1,186	39.9	<0.01
High	1,888	53.6	1,786	60.1	
**Ideal CVH at Exam 5**					
Poor (0–7)	1,197	34.0			
Intermediate (8–11)	2,133	60.6			
Ideal (12–14)	190	5.4			

* *Pearson's χ^2^-test or Wilcoxon-Mann-Whitney test*.

† *Tertiles not 33% due to ties at boundaries and no participants with the same values were included in different tertiles*.

‡ *Median (25th percentile-75th percentile)*.

§ *Due to harmonization of different self-rated health measures across JHS, MESA, and MASALA cohort studies, a binary variable for self-rated health was used to indicate “Good” and “Not good” categories*.

*CVD, cardiovascular disease; CVH, cardiovascular health; MESA, Multi-Ethnic Study of Atherosclerosis*.

Table 2 shows the unadjusted and adjusted risk ratios (aRRs) for the overall relationship between optimism at Exam 2 and the primary outcome, LS7 at Exam 5 in MESA. The aRR comparing high to low optimism was 1.10 (1.04–1.16) and the aRR comparing medium to low optimism was 1.05 (0.99–1.11). Table 3 shows the adjusted relationship between optimism and the primary outcome by level of psychosocial risk, i.e., the aRRs represent the relationship between optimism and LS7 in each psychosocial risk category in MESA. Although psychosocial risk measures largely did not show clear evidence for effect measure modification, there was some evidence for effect modification by neighborhood deprivation. Specifically, participants were more likely to achieve ideal or intermediate CVH with high optimism if they lived in neighborhoods with high- [1.15 (1.02–1.29)] or medium-level deprivation [1.18 (1.08–1.29)]. Strong evidence for the benefits for high optimism was not observed among participants who lived in neighborhoods with low neighborhood deprivation (aRR: 1.00, 95% CI: 0.93–1.08). The point estimates by education, income, and neighborhood safety suggested that participants with lower education, the lowest income, and a not safe neighborhood were more likely to have ideal or intermediate CVH with high optimism. However, there was considerable overlap in the corresponding 95% CIs.

**Table 2:** Risk ratios (RR) for ideal or intermediate vs. poor CVH assessed at exam 5 by optimism levels at exam 2 among MESA participants (*N* = 3,520).

**Outcome**	**High (vs. low) optimism RR (95% CI)**		**Medium (vs. low) optimism RR (95% CI)**	
	**Unadjusted**	Adjusted^*^	**Unadjusted**	Adjusted^*^
Ideal or intermediate vs. poor CVH	1.10 (1.03–1.17)	1.10 (1.04–1.16)	1.08 (1.02–1.15)	1.05 (0.99–1.11)

*Clustering of observations by neighborhood was used in each outcome model (71)*.

* *Adjusted for age, sex, race, nativity, geographic region, marital status, self-rated health, insurance, family CVD history, religiosity, social support, neighborhood social cohesion, education, income, employment, anger, depression, chronic stress, discrimination, neighborhood deprivation, and neighborhood safety*.

**Table 3:** Assessment of effect measure modification of adjusted risk ratios^*^ (aRR) for the relationship between optimism at exam 2 and ideal or intermediate vs. poor CVH at exam 5 by levels of psychosocial risk measures among MESA participants included in the primary analysis sample.

**Psychosocial risk measure (Potential effect measure modifier)**	**Optimism levels (*N =* 3,520)**			**High vs. low optimism: aRR for ideal or intermediate CVH by psychosocial risk levels**		**Medium vs. low optimism: aRR for ideal or intermediate CVH by psychosocial risk levels**		***p* ^†^**
	**Low**	**Medium**	**High**	**aRR**	**95% CI**	**aRR**	**95% CI**	
**Education at Exam 1**								
College degree or more	442	514	451	1.03	(0.95–1.11)	1.03	(0.96–1.10)	0.29
High school or some college	693	496	448	1.17	(1.06–1.28)	1.07	(0.97–1.18)	
Less than high school	210	120	146	1.15	(0.95–1.38)	1.03	(0.84–1.27)	
**Employment at Exam 1**								
Employed	735	638	566	1.11	(1.04–1.20)	1.07	(0.99–1.14)	0.82
Unemployed	610	492	479	1.08	(0.99–1.18)	1.03	(0.94–1.13)	
**Income at Exam 1**								
$50,000+	517	551	515	1.09	(1.01–1.17)	1.03	(0.96–1.11)	0.51
$20,000–$49,999	559	405	335	1.07	(0.97–1.19)	1.09	(0.99–1.20)	
$0–$19,999	269	174	195	1.18	(1.02–1.36)	1.03	(0.88–1.21)	
**Anger at Exam 1**								
Low	408	416	546	1.14	(1.04–1.24)	1.07	(0.96–1.18)	0.91
Medium	460	425	321	1.09	(0.99–1.20)	1.05	(0.96–1.15)	
High	477	289	178	1.06	(0.94–1.19)	1.05	(0.95–1.15)	
**Depression at Exam 1**								
No	1,082	1,044	977	1.11	(1.04–1.18)	1.06	(1.00–1.13)	0.70
Yes	263	86	68	1.02	(0.82–1.28)	0.98	(0.80–1.20)	
**Chronic stress at Exam 1**								
Low	591	558	580	1.13	(1.04–1.22)	1.06	(0.98–1.14)	0.92
Medium	322	284	255	1.06	(0.94–1.19)	1.04	(0.94–1.16)	
High	432	288	210	1.08	(0.95–1.23)	1.05	(0.93–1.19)	
**Discrimination at Exam 1**								
Low	325	311	390	1.15	(1.04–1.27)	1.01	(0.91–1.13)	0.42
Medium	490	434	394	1.05	(0.95–1.17)	1.08	(0.97–1.19)	
High	530	385	261	1.10	(1.00–1.22)	1.05	(0.96–1.16)	
**Neighborhood deprivation at Exam 1**								
Low	452	423	367	1.00	(0.93–1.08)	1.01	(0.94–1.08)	0.06
Medium	490	382	321	1.18	(1.08–1.29)	1.13	(1.04–1.24)	
High	403	325	357	1.15	(1.02–1.29)	1.02	(0.89–1.15)	
**Neighborhood safety at Exam 1**								
Safe	1,101	982	882	1.08	(1.02–1.15)	1.05	(0.99–1.11)	0.44
Not safe	244	148	163	1.19	(1.03–1.38)	1.05	(0.89–1.25)	

*Clustering of observations by neighborhood was used in each outcome model (71)*.

* *Adjusted for age, sex, race, nativity, geographic region, marital status, self-rated health, insurance, family CVD history, religiosity, social support, neighborhood social cohesion, education, income, employment, anger, depression, chronic stress, discrimination, neighborhood deprivation, and neighborhood safety*.

† *P-values were obtained from a global chi-squared test to examine whether at least one of the product term coefficients between optimism and psychosocial risk was different from zero*.

### Secondary Analyses

**Figure 2:**
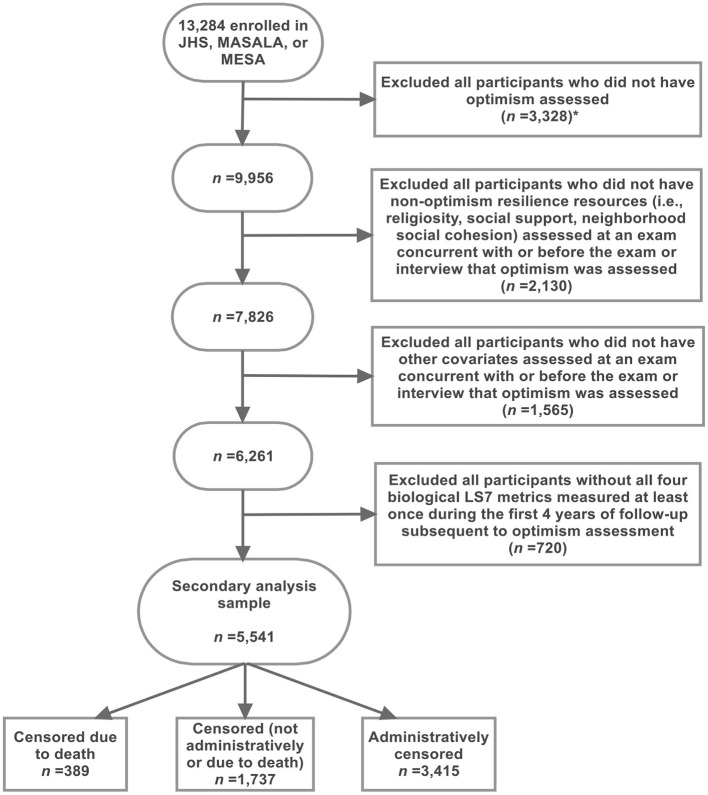
Exclusion criteria applied to identify 5,541 JHS and MESA participants who were included in the secondary analysis sample. The CVH outcome measure was defined using four biological Life's Simple 7 (LS7) metrics that were assessed at exams subsequent to optimism assessment in both the JHS and MESA study. *This exclusion step resulted in the exclusion of all MASALA participants because optimism was not assessed in MASALA. CVH, cardiovascular health; JHS, Jackson Heart Study; LS7, Life's Simple 7; MASALA, Mediators of Atherosclerosis Among South Asians Living in America; MESA, Multi-Ethnic Study of Atherosclerosis.

In the secondary analyses, 5,541 participants were included from JHS and MESA studies (Figure 2). Table 4 shows the demographic characteristics and distributions of optimism, psychosocial risk, and resilience measures comparing the included and a subset of the excluded participants at the relevant interview or relevant exams. In general, comparisons between the included and excluded participants in the secondary analysis sample were similar to the primary analysis sample. However, in this analytic sample, the included participants were older than the excluded participants (median age: 60 vs. 58 years), and most included participants were African American (37.2%). Additionally, 0.7% of the included participants self-reported a history of CVD and stroke.

**Table 4:** Characteristics at the exam or interview that optimism was assessed or exams concurrent with or before optimism assessment comparing the included and a subset of the excluded MESA and JHS participants (i.e., participants without all four biological LS7 metrics assessed at least once during the first 4 years of follow-up subsequent to optimism assessment) in the secondary analyses.

**Characteristics**	**Included**		**Excluded**		*P*-value^*^
	**(*****n*** **=** **5,541)**		**(*****n*** **=** **720)**		
	***N***	**%**	***N***	**%**	
**Optimism**^†^ **at MESA Exam 2 or JHS AF2 Interview**					
High	1,686	30.4	203	28.2	0.37
Medium	1,819	32.8	252	35.0	
Low	2,036	36.7	265	36.8	
**Age**^‡^ **(years) at Exam 1**	60 (52–68)		58 (48–65)		<0.01
**Sex at Exam 1**					
Female	3,051	55.1	454	63.1	<0.01
Male	2,490	44.9	266	36.9	
**Self-reported race/ethnicity at Exam 1**					
White non-Hispanic	1,872	33.8	29	4.0	<0.01
Asian	504	9.1	14	1.9	
African American	2,062	37.2	662	91.9	
Hispanic	1,103	19.9	15	2.1	
**Nativity at Exam 1**					
Other	1,422	25.7	34	4.7	<0.01
U.S.-born	4,119	74.3	686	95.3	
**Region at Exam 1**					
West	924	16.7	13	1.8	<0.01
South	1,618	29.2	645	89.6	
Midwest	1,598	28.8	15	2.1	
Northeast	1,401	25.3	47	6.5	
**Marital status at Exam 1**					
Never married, separated/divorced, widowed	2,102	37.9	306	42.5	0.02
Married	3,439	62.1	414	57.5	
**Self-rated health^§^** **at Exam 1**					
Not good	568	10.3	182	25.3	<0.01
Good	4,973	89.8	538	74.7	
**Health insurance at Exam 1**					
None	486	8.8	86	11.9	<0.01
Public or Private	5,055	91.2	634	88.1	
**Self-history of CVD and stroke at Exam 1**					
No	5,501	99.3	663	92.1	<0.01
Yes	40	0.7	57	7.9	
**Family history of CVD and stroke at Exam 1**					
No	2,387	43.1	291	40.4	0.17
Yes	3,154	56.9	429	59.6	
**Education at Exam 1**					
Less than high school	826	14.9	99	13.8	0.11
High school or some college	2,583	46.6	315	43.8	
College degree or more	2,132	38.5	306	42.5	
**Employment at Exam 1**					
Unemployed	2,641	47.7	354	48.8	0.58
Employed	2,900	52.3	369	51.3	
**Income at Exam 1**					
$0–$19,999	1,197	21.6	182	25.3	0.04
$20,000–$49,999	2,023	36.5	265	36.8	
$50,000+	2,321	41.9	273	37.9	
**Anger**^†^ **at Exam 1**					
Low	2,170	39.2	234	32.5	<0.01
Medium	1,810	32.7	185	25.7	
High	1,561	28.2	301	41.8	
**Depression at Exam 1**					
No	4,787	86.4	576	80.0	<0.01
Yes	754	13.6	144	20.0	
**Chronic stress**^†^ **at Exam 1**					
Low	2,458	44.4	147	20.4	<0.01
Medium	1,847	33.3	297	41.3	
High	1,236	22.3	276	38.3	
**Discrimination**^†^ **at Exam 1**					
Low	2,053	37.1	190	26.4	<0.01
Medium	1,848	33.4	216	30.0	
High	1,640	29.6	314	43.6	
**Neighborhood deprivation**^†^ **at Exam 1**					
Low	2,150	38.8	152	21.1	<0.01
Medium	1,842	33.2	208	28.9	
High	1,549	28.0	360	50.0	
**Neighborhood safety at Exam 1**					
Safe	4,491	81.1	445	61.8	<0.01
Not safe	1,050	19.0	275	38.2	
**Religiosity at MESA Exam 2 or JHS Exam 1**					
Not high	2,782	50.2	276	38.3	<0.01
High	2,759	49.8	444	61.7	
**Social support at Exam 1**					
Not high	1,976	35.7	211	29.3	<0.01
High	3,565	64.3	509	70.7	

* *Pearson's χ^2^-test or Wilcoxon-Mann-Whitney test*.

† *Tertiles not 33% due to ties at boundaries and no participants with the same values were included in different tertiles*.

‡ *Median (25th percentile-75th percentile)*.

§ *Due to the harmonization of different self-rated health measures across JHS, MESA, and MASALA cohort studies, a binary variable for self-rated health was used to indicate “Good” and “Not good” categories*.

*CVD, cardiovascular disease; AF2, Second Annual Follow-up; JHS, Jackson Heart Study; MESA, Multi-Ethnic Study of Atherosclerosis*.

Table 5 shows the unadjusted and adjusted RRs for the overall relationship between optimism and the secondary outcomes using the four biological LS7 metrics. Without the optimism-visit product terms, the aRR (95% CI) for having ideal or intermediate (no poor) metrics compared to having at least 1 poor metric among those with high optimism (vs. low) was 1.05 (0.98–1.12) and the aRR (95% CI) among those with medium optimism (vs. low) was 1.04 (0.97–1.11). The aRR for having a lower cardiovascular risk as a function of high optimism was 1.01 (0.98–1.03), and for medium optimism, the corresponding aRR was 1.01 (0.98–1.03).

**Table 5:** Risk ratios (RR) for ideal or intermediate (no poor) metrics vs. at least 1 poor metric and RR for lower cardiovascular risk (0–1 poor metrics) vs. non-lower cardiovascular risk (2–4 poor metrics) by optimism levels at exam 2 in MESA and the second annual follow-up interview in JHS using four biological life's simple 7 measures (BMI, blood pressure, cholesterol, and glucose) assessed during follow-up subsequent to optimism assessment among MESA and JHS participants included in the secondary analysis sample (*N* = 5,541).

**Outcome**	**Optimism-visit product term in outcome model**		**High vs. low optimism RR (95% CI)**		**Medium vs. low optimism RR (95% CI)**	
			**Unadjusted**	**Adjusted^‡^**	**Unadjusted**	**Adjusted^‡^**
Ideal or intermediate (no poor) metrics vs. 1 or more poor metrics	No optimism-visit product term		1.02 (0.95–1.10)	1.05 (0.98–1.12)	1.03 (0.95–1.10)	1.04 (0.97–1.11)
	Optimism-visit product term is present	Visit 1^*^	1.02 (0.95–1.10)	1.04 (0.97–1.12)	1.05 (0.97–1.13)	1.05 (0.98–1.13)
		Visit 2^*^	1.02 (0.92–1.13)	1.06 (0.96–1.16)	0.99 (0.90–1.09)	1.01 (0.92–1.11)
Lower cardiovascular risk (0–1 poor metrics) vs. non-lower cardiovascular risk (2–4 poor metrics)	No optimism-visit product term		1.00 (0.98–1.03)	1.01 (0.98–1.03)	1.01 (0.98–1.03)	1.01 (0.98–1.03)
	Optimism-visit product term is present	Visit 1^†^	1.02 (0.99–1.05)	1.02 (0.99–1.05)	1.00 (0.97–1.03)	1.00 (0.97–1.03)
		Visit 2^†^	0.98 (0.94–1.02)	0.98 (0.95–1.02)	1.01 (0.98–1.05)	1.02 (0.98–1.05)

*Clustering of observations by neighborhood was used in each repeated-measures modified Poisson regression model. Clustering by neighborhood should also account for within subject correlation in the outcome when subjects are nested within neighborhoods (76). Clustering by subject did not change inference*.

* *Optimism-visit product term coefficients for unadjusted model: −0.06, 0.0007, p = 0.40; adjusted model: −0.04, 0.02, p = 0.48*.

† *Optimism-visit product term coefficients for unadjusted model: 0.01, −0.04, p = 0.03; adjusted model: 0.02, −0.04, p = 0.03*.

‡ *Adjusted for visit, age, sex, race, nativity, geographic region, marital status, self-rated health, insurance, self-history of CVD, family CVD history, religiosity, social support, education, income, employment, anger, depression, chronic stress, discrimination, neighborhood deprivation, and neighborhood safety*.

For analyses concerning the overall relationship between optimism and the secondary outcomes that include the optimism-visit product terms, the aRRs are presented by visit in Table 5. For both secondary outcomes, the aRRs with and without the optimism-visit product term did not differ meaningfully. Also, for both secondary outcomes, the aRRs from the models with the optimism-visit product terms did not differ meaningfully across the two visits. Therefore, the remainder of the results section and the discussion pertaining to the secondary analyses primarily focus on the results without the optimism-visit product term (Tables 5–**7**). However, secondary analysis results that are stratified by level of psychosocial risk and include the optimism-visit product term are included in the supplement (Supplementary Tables 1, 2). The secondary analysis results that are stratified by level of psychosocial risk and include the optimism-visit product term are largely consistent with and do not differ meaningfully from the corresponding stratified results that exclude the optimism-visit product term.

Table 6 shows some evidence of heterogeneity for the ideal or intermediate (no poor) metrics vs. at least 1 poor metric outcome. Low or medium chronic stress exposure was compatible with a positive relationship between medium optimism and the outcome [aRR: 1.06, 95% CI: 0.96–1.16 and 1.10 (0.99–1.22), respectively]. However, exposure to high chronic stress suggested compatibility with a negative relationship between medium optimism and the outcome (aRR: 0.89, 95% CI: 0.76–1.05) (Table 6).

**Table 6:** Assessment of effect measure modification of adjusted risk ratios^*^ (aRR) for the relationship between optimism at exam 2 in MESA and the second annual follow-up interview in JHS and ideal or intermediate (no poor) metrics vs. at least 1 poor metric using four biological Life's Simple 7 metrics (BMI, blood pressure, cholesterol, and glucose) by levels of psychosocial risk measures among MESA and JHS participants included in the secondary analysis sample (*N* = 5,541).

**Psychosocial risk measure (Potential effect measure modifier)**	**High vs. low optimism: aRR for ideal or intermediate (no poor) metrics by psychosocial risk levels**		**Medium vs. low optimism: aRR for ideal or intermediate (no poor) metrics by psychosocial risk levels**		***p* ^†^**
	**aRR**	**95% CI**	**aRR**	**95% CI**	
**Education at Exam 1**					
College degree or more	1.01	(0.91–1.11)	1.01	(0.92–1.10)	0.73
High school or some college	1.09	(0.98–1.21)	1.04	(0.93–1.16)	
Less than high school	1.05	(0.87–1.28)	1.12	(0.93–1.34)	
**Employment at Exam 1**					
Employed	1.06	(0.97–1.17)	1.03	(0.94–1.13)	0.83
Unemployed	1.03	(0.93–1.14)	1.04	(0.94–1.15)	
**Income at Exam 1**					
$50,000+	1.10	(1.00–1.21)	1.09	(1.00–1.19)	0.14
$20,000–$49,999	0.93	(0.83–1.05)	0.97	(0.87–1.10)	
$0–$19,999	1.14	(0.97–1.33)	1.03	(0.88–1.21)	
**Anger at Exam 1**					
Low	1.07	(0.95–1.20)	1.07	(0.94–1.21)	0.92
Medium	1.05	(0.94–1.18)	1.05	(0.95–1.17)	
High	1.02	(0.89–1.16)	0.98	(0.87–1.11)	
**Depression at Exam 1**					
Low	1.05	(0.98–1.13)	1.03	(0.95–1.11)	0.49
High	0.96	(0.75–1.23)	1.12	(0.91–1.37)	
**Chronic stress at Exam 1**					
Low	1.09	(0.99–1.21)	1.06	(0.96–1.16)	0.11
Medium	0.98	(0.87–1.11)	1.10	(0.99–1.22)	
High	1.04	(0.88–1.23)	0.89	(0.76–1.05)	
**Discrimination at Exam 1**					
Low	1.00	(0.90–1.12)	1.01	(0.90–1.13)	0.59
Medium	1.07	(0.95–1.20)	1.09	(0.98–1.22)	
High	1.10	(0.95–1.27)	1.00	(0.88–1.14)	
**Neighborhood deprivation at Exam 1**					
Low	1.00	(0.90–1.10)	1.03	(0.94–1.14)	0.67
Medium	1.07	(0.95–1.21)	1.04	(0.92–1.17)	
High	1.11	(0.96–1.28)	1.03	(0.88–1.21)	
**Neighborhood safety at Exam 1**					
Safe	1.04	(0.97–1.12)	1.04	(0.97–1.12)	0.81
Not safe	1.08	(0.90–1.29)	1.01	(0.85–1.20)	

*Clustering of observations by neighborhood was used in each outcome model. Clustering by neighborhood should also account for within subject correlation in the outcome when subjects are nested within neighborhoods (76). Clustering by subject did not change inference*.

* *Adjusted for visit, age, sex, race, nativity, geographic region, marital status, self-rated health, insurance, self-history of CVD, family CVD history, religiosity, social support, education, income, employment, anger, depression, chronic stress, discrimination, neighborhood deprivation, and neighborhood safety*.

† *P-values were obtained from a global chi-squared test to examine whether at least one of the product term coefficients between optimism and psychosocial risk was different from zero*.

For lower cardiovascular risk outcomes, we observed largely null relationships and there was little evidence for effect modification by psychosocial risks (Table 7). Visual illustrations of all of the previously described effect measure modification results (Tables 3, 6, 7) are provided as plots in Supplementary Figures 1–3.

**Table 7:** Assessment of effect measure modification of adjusted risk ratios^*^ (aRR) for the relationship between optimism at exam 2 in MESA and the second annual follow-up interview in JHS and lower cardiovascular risk (0–1 poor metrics) compared with non-lower cardiovascular risk (2–4 poor metrics) using the four biological Life's Simple 7 metrics (BMI, blood pressure, cholesterol, and glucose) by levels of psychosocial risk measures among MESA and JHS participants included in the secondary analysis sample (*N* = 5,541).

**Psychosocial risk measure (Potential effect measure modifier)**	**High vs. low optimism: aRR for lower CV risk (0–1 poor metrics) by psychosocial risk levels**		**Medium vs. low optimism: aRR for lower CV risk (0–1 poor metrics) by psychosocial risk levels**		***p* ^†^**
	**aRR**	**95% CI**	**aRR**	**95% CI**	
**Education at Exam 1**					
College degree or more	1.01	(0.97–1.04)	1.02	(0.99–1.05)	0.91
High school or some college	1.01	(0.97–1.05)	1.00	(0.96–1.04)	
Less than high school	1.00	(0.93–1.08)	1.01	(0.94–1.09)	
**Employment at Exam 1**					
Employed	1.02	(0.98–1.05)	1.02	(0.99–1.06)	0.39
Unemployed	0.99	(0.96–1.03)	0.99	(0.95–1.03)	
**Income at Exam 1**					
$50,000+	1.04	(1.00–1.07)	1.04	(1.00–1.07)	0.24
$20,000–$49,999	0.98	(0.93–1.02)	0.98	(0.94–1.02)	
$0–$19,999	1.00	(0.94–1.06)	1.00	(0.94–1.06)	
**Anger at Exam 1**					
Low	1.01	(0.97–1.05)	1.03	(0.99–1.08)	0.50
Medium	0.99	(0.95–1.04)	1.00	(0.96–1.04)	
High	1.03	(0.98–1.08)	0.99	(0.94–1.04)	
**Depression at Exam 1**					
Low	1.00	(0.98–1.03)	1.01	(0.98–1.04)	0.63
High	1.04	(0.96–1.13)	0.99	(0.92–1.07)	
**Chronic stress at Exam 1**					
Low	1.00	(0.97–1.04)	1.01	(0.97–1.04)	0.61
Medium	1.02	(0.98–1.07)	1.04	(0.99–1.08)	
High	0.99	(0.92–1.06)	0.97	(0.91–1.03)	
**Discrimination at Exam 1**					
Low	1.00	(0.96–1.04)	1.02	(0.98–1.07)	0.15
Medium	0.99	(0.95–1.04)	1.01	(0.97–1.05)	
High	1.04	(0.99–1.10)	0.99	(0.94–1.04)	
**Neighborhood deprivation at Exam 1**					
Low	1.01	(0.97–1.04)	1.02	(0.99–1.05)	0.87
Medium	1.01	(0.97–1.06)	1.00	(0.96–1.04)	
High	1.00	(0.94–1.06)	1.01	(0.95–1.07)	
**Neighborhood safety at Exam 1**					
Safe	1.00	(0.97–1.03)	1.01	(0.99–1.04)	0.27
Not safe	1.03	(0.96–1.11)	0.99	(0.92–1.06)	

*Clustering of observations by neighborhood was used in each outcome model. Clustering by neighborhood should also account for within subject correlation in the outcome when subjects are nested within neighborhoods (76). Clustering by subject did not change inference*.

* *Adjusted for visit, age, sex, race, nativity, geographic region, marital status, self-rated health, insurance, self-history of CVD, family CVD history, religiosity, social support, education, income, employment, anger, depression, chronic stress, discrimination, neighborhood deprivation, and neighborhood safety*.

† *P-values were obtained from a global chi-squared test to examine whether at least one of the product term coefficients between optimism and psychosocial risk was different from zero*.

### Sensitivity Analyses

Our sensitivity analysis did not observe any change to the aRRs in our primary analyses when we also controlled for follow-up time since the exposure assessment (not reported). Our findings did not change in the secondary analyses when we clustered by subject (not reported). Further, using an exchangeable working correlation structure did not change inferences in our primary or secondary analyses (not reported).

When we stratified by cohort in our secondary analyses without the optimism-visit product terms, the aRRs for high or medium optimism were similar in both the JHS and MESA cohorts but were slightly stronger in MESA compared to the JHS cohort (Table 8). With the inclusion of optimism-visit product terms, the association for ideal or intermediate (no poor) metrics with high optimism seemed to be stronger at visit 2 compared to visit 1 in both cohorts. In JHS, the association was reversed in visit 2 compared to visit 1. For medium optimism in JHS, the association for ideal or intermediate (no poor) metrics was

**Table 8:** Cohort-stratified adjusted^*^ risk ratios (aRR) for ideal or intermediate (no poor) metrics vs. at least 1 poor metric and aRR for lower cardiovascular risk (0–1 poor metrics) vs. non-lower cardiovascular risk (2–4 poor metrics) by optimism levels at exam 2 in MESA and the second annual follow-up interview in JHS using four biological Life's Simple 7 measures (BMI, blood pressure, cholesterol, and glucose) assessed during follow-up subsequent to optimism assessment.

**Outcome**	**Optimism-visit product term in outcome model**		**High vs. low optimism aRR (95% CI)**		**Medium vs. low optimism aRR (95% CI)**	
			**JHS**	**MESA**	**JHS**	**MESA**
			**(*N* = 851)**	**(*N* = 4,690)**	**(*N* = 851)**	**(*N* = 4,690)**
Ideal or intermediate (no poor) metrics vs. 1 or more poor metrics	No optimism-visit product term		1.02 (0.82–1.26)	1.05 (0.98–1.13)	1.00 (0.77–1.30)	1.04 (0.97–1.11)
	Optimism-visit product term is present	Visit 1^†^	0.99 (0.81–1.22)	1.04 (0.97–1.12)	0.93 (0.70–1.23)	1.06 (0.99–1.14)
		Visit 2^†^	1.06 (0.77–1.45)	1.07 (0.97–1.18)	1.11 (0.79–1.55)	1.00 (0.91–1.10)
Lower cardiovascular risk (0–1 poor metrics) vs. non–lower cardiovascular risk (2–4 poor metrics)	No optimism-visit product term		0.96 (0.87–1.05)	1.01 (0.99–1.04)	0.96 (0.89–1.05)	1.01 (0.99–1.04)
	Optimism-visit product term is present	Visit 1^‡^	0.99 (0.89–1.09)	1.02 (0.99–1.05)	0.93 (0.85–1.02)	1.01 (0.98–1.04)
		Visit 2^‡^	0.93 (0.82–1.04)	1.00 (0.96–1.03)	1.01 (0.90–1.12)	1.02 (0.98–1.05)

*Clustering of observations by neighborhood was used in each outcome model. Clustering by neighborhood should also account for within subject correlation in the outcome when subjects are nested within neighborhoods (76). Clustering by subject did not change inference*.

* *Adjusted for visit, age, sex, race, nativity, geographic region, marital status, self-rated health, insurance, self-history of CVH, family CVD history, religiosity, social support, education, income, employment, anger, depression, chronic stress, discrimination, neighborhood deprivation, and neighborhood safety. In JHS, race, nativity, and geographic region were excluded from the model because all participants were African American residing in one geographic region. In MESA, self-history of CVD was excluded from the model because all participants were free of CVD at study enrollment*.

† *Optimism-visit product term coefficients for adjusted model in JHS: 0.18, 0.06, p = 0.52; in MESA: −0.06, 0.03, p = 0.21; p-value obtained from global chi-squared test*.

‡ *Optimism-visit product term coefficients for adjusted model in JHS 0.07, −0.06, p = 0.07; in MESA: 0.01, −0.03, p = 0.23; p-value obtained from global chi-squared test*.

reversed in visit 2 compared to visit 1, and in MESA, the association was positive at visit 1 but null at visit 2. For the lower cardiovascular risk outcome, the relationship did not differ meaningfully by visit in MESA. However, the aRRs were reversed in visit 2 compared to visit 1 in JHS for medium optimism.

## Discussion

The present study utilized harmonized data from two U.S.-based cardiovascular cohorts (MESA and JHS) to examine the relationship between optimism, psychosocial risk factors, and CVH. The results from the primary analyses in MESA participants provided evidence that those who report a higher level of optimism compared to a lower level were more likely to have ideal or intermediate CVH. However, participants who lived in neighborhoods with low neighborhood deprivation did not seem to benefit from high optimism, which provided some evidence of effect measure modification by psychosocial risk. Our secondary analyses that included MESA and JHS participants showed similar or less compelling evidence for a positive association between higher levels of optimism and CVH and for effect measure modification.

Our findings suggesting that high optimism is beneficial for CVH is supported by prior studies. Past cross-sectional studies, including studies from the JHS and MESA cohorts, have indicated that participants with high optimism were more likely to have better CVH outcomes (38–40). Moreover, there is increasing evidence that suggests high optimism leads to better health behaviors and CVD outcomes (81), such as in a meta-analysis showing the positive association between high optimism and better CVD-related outcomes (82). Further, optimism was associated with higher LS7 scores over time among young adults in the U.S. (41). These findings, including our results, may be due to both physiological and behavioral mechanisms by which optimism may influence CVH. The physiological mechanism may involve the processes such as reduced inflammation, and the behavioral mechanism may involve optimistic individuals engaging in more physical activities, eating a healthier diet, and/or not smoking, as well as using adaptive coping strategies against stress (33, 42, 83). Hence, there is now a growing pool of evidence supporting a positive association between higher optimism and better CVH outcomes, including evidence based on prospective analyses.

We hypothesized that individuals with the greatest exposure to psychosocial risks would benefit the most from higher levels of resilience resources. In our study, neighborhood deprivation and chronic stress demonstrated some evidence of effect measure modification for certain CVH outcomes (i.e., ideal or intermediate CVH using LS7 and ideal or intermediate (no poor) metrics based on four biological LS7 metrics). Specifically, high optimism only appeared to be beneficial for participants who lived in neighborhoods with higher neighborhood deprivation. In contrast, only participants who had lower exposure to chronic stress (as measured by self-report) benefitted from medium optimism. Although the present study findings for chronic stress were inconsistent with our hypothesis, the concept of allostatic load may explain the apparent inconsistency. In short, the repeated activation of physiological systems designed to maintain homeostasis of the body (e.g., cardiovascular system, sympathetic nervous system) during repeated stressor exposures may lead to dysregulation (e.g., fail to activate) when the stressors are chronic (84). Living in neighborhoods with high deprivation is also considered a chronic stressor, but it is plausible that people living in neighborhoods with high deprivation may have access to built environment resources, such as access to healthy foods or physical activity resources, which may favorably support healthy behaviors. Further, the Reserve Capacity Model posits that individuals in low SEP are exposed to more adversities or chronic stress exposures (33), have higher allostatic load and experience over-response to these chronic stressors (85). Consequently, the chronic activation of these physiological systems may be too substantial to be offset by higher levels of optimism or other resilience resources (34, 86). However, despite the apparent inconsistency concerning chronic stress, our study findings regarding higher neighborhood deprivation were consistent with our hypothesis. Although compelling evidence for effect modification of the relationship between optimism and CVH outcomes was not observed for other psychosocial risks, this lack of evidence may partly be due to random error. Hence, effect modification by psychosocial risks, particularly by self-reported chronic stress and objectively measured neighborhood deprivation, warrants further study.

The findings from our study may have practical implications for optimism-based interventions aimed at reducing and preventing adverse CVH outcomes. Despite some research considering optimism as an unmodifiable trait (87), optimistic mindsets may be induced by intervention or practice via broadly termed “cognitive-behavioral therapies” (CBT) (87–89). In general, there is a lack of optimism-based intervention studies in populations with and without CVD, but existing CBT interventions have shown to improve short-term optimism (83). Another possible strategy to improve optimism is the “Best Possible Self” (BPS) intervention, which is based on the positive writing paradigm, i.e., patients/clients write about the positive topics, such as focusing on their best possible future, that improves their positive mood and psychological well-being (90). Two meta-analytic studies have shown that BPS interventions could effectively improve optimism compared to controls (91, 92). In addition, interventions on positive psychological well-being have demonstrated favorable health behaviors, such as increased physical activity (93, 94). For example, the positive psychology intervention group had an increased odds of achieving higher physical activity levels than the control group (odds ratio = 1.74) (93), while another study showed a moderate effect size (Cohen's *d* = 1.19, *p* = 0.007) for improvements in healthy behavior adherence that included physical activity and diet among those in the intervention group compared to controls (94); however, there is varying evidence on the biological effects (95). Some trials targeting optimism have also shown improvements in psychosocial risk exposures, such as reduced depressive symptoms (96). Hence, optimism-focused interventions may improve optimism, reduce psychosocial risks and have beneficial CVH outcomes. Therefore, our findings concerning effect measure modification may help to identify subgroups who would benefit most from interventions targeted at building optimism to improve CVH. For example, individuals who live in neighborhoods with higher neighborhood deprivation may benefit from interventions to improve optimism, while individuals who live in neighborhoods with low neighborhood deprivation may not.

Although some of our findings are compelling, there are several limitations to our study. Psychometric analyses (i.e., confirmatory factor analyses and reliability analyses) were conducted on the harmonized JHS, MESA, and MASALA cohort data for each of the various resilience and psychosocial risk measures used in the study. In general, no measure displayed each of three ideal psychometric characteristics (excellent model fit, high item loadings, and high internal consistency reliability) across the harmonized JHS, MESA, and MASALA cohorts and also within each cohort. Although some measures did display relatively better psychometric characteristics than others, there was general concern about the overall quality of the resilience and psychosocial risk measures. Another limitation of harmonization was that we could only use the data available in the cohorts. For example, self-rated health was measured using different assessment tools in JHS and MESA studies, and we dichotomized the variable into good and not good to harmonize the measure. Similarly, all of the LS7 metrics and neighborhood social cohesion were only available to be used, or able to be controlled for, in the primary analyses which may explain some of the observed differences in the findings between the primary and secondary analyses. In addition, there may be residual confounding or selection bias or measurement error, especially given that many measures were self-reported. Further, there may be model misspecification of our outcome regression models (e.g., the relationship between continuous covariate and outcome is not completely accurate even with the use of restricted quadratic splines) (72, 97). Lastly, we may not have been adequately powered to explore effect measure modification.

Despite these limitations, our study has several strengths. First, to the best of our knowledge, our study is the first to examine effect measure modification by levels of psychosocial risks in the relationship between optimism and CVH outcomes in racially/ethnically diverse populations, i.e., among African American, White non-Hispanic, Asian, and Hispanic adults from the MESA and JHS study. In the comparison between the two cohort studies in our secondary analyses, we showed that the findings from the data from the JHS participants, who were all African American adults, were similar to the findings from the data from MESA participants. However, the relationship was weaker among the JHS participants, and even reversed in the analyses by visit. This weakening or reversal in the relationship between optimism and CVH outcomes may have been due to random error because of a smaller sample size in JHS. Second, utilizing harmonized data allowed for increased power to examine the relationship between optimism and CVH outcomes and for comparability between different cohorts. Third, we used prospective observational cohort data to establish temporality. Last, we used a modified Poisson regression model fit with generalized estimating equations to estimate RRs and obtain robust variances that account for clustered data (71).

The present study provided some evidence of a positive relationship between higher optimism and better CVH using LS7 metrics in two U.S.-based cohorts. However, we did not find clear evidence for a relationship between optimism and lower cardiovascular risk (secondary analysis). Our study could not utilize more metrics in the secondary analyses because dietary intake and physical activity were not assessed in all follow-up exams. However, some of the findings from this study suggest that optimism is a promising area for intervention to improve CVH, and prevent and reduce adverse CVD outcomes. Further prospective studies are needed to assess the beneficial effects of optimism-based interventions on CVH, especially in a diverse population in the presence of varying levels of psychosocial risks.

## Data Availability Statement

The datasets presented in this article are not readily available because the data may be available by request and with permission from the study sites at JHS, MASALA, and MESA. Requests to access the datasets should be directed to JHS (https://www.jacksonheartstudy.org/Research/Study-Data/Data-Access), MASALA (https://www.masalastudy.org/for-researchers), and MESA (https://www.mesa-nhlbi.org/Publications.aspx).

## Ethics Statement

The studies involving human participants were reviewed and approved by the Institutional Review Boards (IRB) at each study sites (JHS, MESA, and MASALA). The secondary analysis of the data analyzed in this paper was approved by the IRB at Brown University (Providence, Rhode Island). The patients/participants provided their written informed consent to participate in this study.

## Author Contributions

AD and CH contributed to the conception and design of the study. AD, LD, and MS organized and harmonized the database. JP performed the statistical analysis with the supervision of CH. JP wrote the first draft of the manuscript. JP, AD, BN, MS, EL, JF, LD, MS, CE, and CH contributed to manuscript revision, read, and approved the submitted version. All authors contributed to the article and approved the submitted version.

## Funding

Research reported in this publication was supported by the National Heart, Lung, and Blood Institute of the National Institutes of Health under Award Number R01HL135200. One hundred percent of the project costs ($438,847) are financed with Federal money. The content is solely the responsibility of the authors and does not necessarily represent the official views of the National Institutes of Health.

The Jackson Heart Study (JHS) was supported and conducted in collaboration with Jackson State University (HHSN268201800013I), Tougaloo College (HHSN268201800014I), the Mississippi State Department of Health (HHSN268201800015I), and the University of Mississippi Medical Center (HHSN268201800010I, HHSN268201800011I and HHSN268201800012I) contracts from the National Heart, Lung, and Blood Institute (NHLBI) and the National Institute on Minority Health and Health Disparities (NIMHD).

The Multi-Ethnic Study of Atherosclerosis (MESA) study was supported by contracts 75N92020D00001, HHSN268201500003I, N01-HC-95159, 75N92020D00005, N01-HC-95160, 75N92020D00002, N01-HC-95161, 75N92020D00003, N01-HC-95162, 75N92020D00006, N01-HC-95163, 75N92020D00004, N01-HC-95164, 75N92020D00007, N01-HC-95165, N01-HC-95166, N01-HC-95167, N01-HC-95168, and N01-HC-95169 from the National Heart, Lung, and Blood Institute, and by grants UL1-TR-000040, UL1-TR-001079, and UL1-TR-001420 from the National Center for Advancing Translational Sciences (NCATS). A full list of participating MESA investigators and institutions can be found at: http://www.mesa-nhlbi.org.

The Mediators of Atherosclerosis in South Asians Living in America (MASALA) project described was supported by Grant Number R01HL093009 from the National Heart, Lung, and Blood Institute and the National Center for Research Resources and the National Center for Advancing Translational Sciences, National Institutes of Health, through UCSF-CTSI Grant Number UL1RR024131.

## Author Disclaimer

The views expressed in this manuscript are those of the authors and do not necessarily represent the views of the National Heart, Lung, and Blood Institute; the National Institutes of Health; or the U.S. Department of Health and Human Services.

## Conflict of Interest

The authors declare that the research was conducted in the absence of any commercial or financial relationships that could be construed as a potential conflict of interest.

## Publisher's Note

All claims expressed in this article are solely those of the authors and do not necessarily represent those of their affiliated organizations, or those of the publisher, the editors and the reviewers. Any product that may be evaluated in this article, or claim that may be made by its manufacturer, is not guaranteed or endorsed by the publisher.
