# Supplementary material for: Examining Optimism, Psychosocial Risks, and Cardiovascular Health Using Life's Simple 7 Metrics in the Multi-Ethnic Study of Atherosclerosis and the Jackson Heart Study
Source: Front Cardiovasc Med. 2021 Dec 15;8:788194. doi: 10.3389/fcvm.2021.788194 (PMC8714850; doi:10.3389/fcvm.2021.788194)
Supplement: Supplementary file 1 [file Table_1.DOCX]

**Supplemental Table 1.** Assessment of effect measure modification of adjusted risk ratios^*^ (aRR) for the relationship between optimism at Exam 2 in MESA and the Second Annual Follow-up Interview in JHS and ideal or intermediate (no poor) metrics versus at least 1 poor metric using four biological Life’s Simple 7 metrics (BMI, blood pressure, cholesterol, and glucose) by visit and levels of psychosocial risk measures among MESA and JHS participants included in the secondary analysis sample (N=5,541).

| **Psychosocial risk measure**  **(Potential effect measure modifier)** | **High versus low optimism:**  **aRR for ideal or intermediate (no poor) metrics by visit and psychosocial risk levels** | | | | **Medium versus low optimism:**  **aRR for ideal or intermediate (no poor) metrics by visit and psychosocial risk levels** | | | | | | **p**^†^ | |
| --- | --- | --- | --- | --- | --- | --- | --- | --- | --- | --- | --- | --- |
|  | **Visit 1** | | **Visit 2** | | **Visit 1** | | **Visit 2** | | | |  | |
|  | **aRR** | **95% CI** | **aRR** | **95% CI** | **aRR** | **95% CI** | **aRR** | | **95% CI** | |  | |
| **Education at Exam 1** |  |  |  |  |  |  |  | |  | |  | |
| College degree or more | 1.02 | (0.91-1.14) | 0.99 | (0.87-1.12) | 1.03 | (0.93-1.13) | 0.97 | | (0.86-1.10) | | 0.80 | |
| High school or some college | 1.05 | (0.94-1.16) | 1.15 | (0.98-1.34) | 1.04 | (0.93-1.16) | 1.04 | | (0.89-1.22) | |  |  |
| Less than high school | 1.08 | (0.88-1.34) | 0.99 | (0.72-1.36) | 1.18 | (0.96-1.45) | 0.99 | | (0.73-1.34) | |  |  |
| **Employment at Exam 1** |  |  |  |  |  |  |  | |  | |  | |
| Employed | 1.08 | (0.98-1.19) | 1.04 | (0.91-1.17) | 1.03 | (0.94-1.13) | 1.03 | | (0.91-1.16) | | 0.29 | |
| Unemployed | 0.99 | (0.89-1.11) | 1.09 | (0.94-1.27) | 1.07 | (0.97-1.19) | 0.99 | | (0.85-1.14) | |  |  |
| **Income at Exam 1** |  |  |  |  |  |  |  | |  | |  | |
| $50,000+ | 1.08 | (0.98-1.20) | 1.13 | (1.00-1.28) | 1.10 | (1.00-1.21) | 1.07 | | (0.95-1.20) | | 0.56 | |
| $20,000-$49,999 | 0.97 | (0.86-1.09) | 0.87 | (0.72-1.04) | 1.00 | (0.89-1.13) | 0.93 | | (0.79-1.10) | |  |  |
| $0-$19,999 | 1.07 | (0.91-1.26) | 1.28 | (1.01-1.62) | 1.03 | (0.87-1.21) | 1.03 | | (0.80-1.34) | |  |  |
| **Anger at Exam 1** |  |  |  |  |  |  |  | |  | |  | |
| Low | 1.01 | (0.90-1.14) | 1.18 | (1.00-1.39) | 1.03 | (0.91-1.17) | 1.14 | | (0.94-1.39) | | 0.82 | |
| Medium | 1.10 | (0.97-1.24) | 0.98 | (0.84-1.15) | 1.09 | (0.98-1.22) | 0.98 | | (0.84-1.14) | |  |  |
| High | 0.99 | (0.85-1.14) | 1.06 | (0.88-1.26) | 1.01 | (0.88-1.15) | 0.94 | | (0.81-1.10) | |  |  |
| **Depression at Exam 1** |  |  |  |  |  |  |  | |  | |  | |
| Low | 1.04 | (0.96-1.12) | 1.07 | (0.97-1.19) | 1.03 | (0.96-1.12) | 1.02 | | (0.92-1.13) | | 0.37 | |
| High | 0.98 | (0.74-1.29) | 0.93 | (0.67-1.30) | 1.19 | (0.96-1.48) | 1.00 | | (0.75-1.33) | |  |  |
| **Chronic stress at Exam 1** |  |  |  |  |  |  |  | |  | |  | |
| Low | 1.09 | (0.99-1.20) | 1.11 | (0.95-1.29) | 1.07 | (0.97-1.18) | 1.03 | | (0.90-1.17) | | 0.01 | |
| Medium | 1.00 | (0.87-1.14) | 0.96 | (0.80-1.15) | 1.15 | (1.03-1.30) | 1.01 | | (0.86-1.17) | |  |  |
| High | 1.00 | (0.84-1.18) | 1.12 | (0.89-1.40) | 0.84 | (0.71-1.00) | 0.98 | | (0.79-1.22) | |  |  |
| **Discrimination at Exam 1** |  |  |  |  |  |  |  | |  | |  | |
| Low | 1.01 | (0.90-1.13) | 0.99 | (0.85-1.16) | 1.03 | (0.91-1.16) | 0.98 | | (0.84-1.15) | | 0.38 | |
| Medium | 1.02 | (0.90-1.15) | 1.15 | (0.97-1.36) | 1.10 | (0.99-1.23) | 1.08 | | (0.91-1.29) | |  |  |
| High | 1.13 | (0.97-1.31) | 1.05 | (0.87-1.27) | 1.02 | (0.88-1.17) | 0.98 | | (0.82-1.16) | |  |  |
| **Neighborhood deprivation at Exam 1** | | |  |  |  |  | |  | |  | |  |
| Low | 0.95 | (0.86-1.06) | 1.07 | (0.94-1.23) | 1.04 | (0.94-1.15) | 1.03 | | (0.91-1.16) | | 0.21 | |
| Medium | 1.09 | (0.96-1.23) | 1.04 | (0.86-1.25) | 1.06 | (0.95-1.20) | 0.99 | | (0.84-1.17) | |  |  |
| High | 1.14 | (0.98-1.32) | 1.05 | (0.85-1.30) | 1.05 | (0.90-1.24) | 0.99 | | (0.78-1.27) | |  |  |
| **Neighborhood safety at Exam 1** | |  |  |  |  |  |  | |  | |  | |
| Safe | 1.04 | (0.96-1.12) | 1.05 | (0.94-1.16) | 1.06 | (0.98-1.14) | 1.01 | | (0.91-1.11) | | 0.79 | |
| Not safe | 1.06 | (0.88-1.28) | 1.11 | (0.87-1.42) | 1.01 | (0.84-1.21) | 1.01 | | (0.80-1.29) | |  |  |

Note: Clustering of observations by neighborhood was used in each outcome model. Clustering by neighborhood should also account for within subject correlation in the outcome when subjects are nested within neighborhoods (76). Clustering by subject did not change inference.

^*^ Adjusted for visit, age, gender, race, nativity, geographic region, marital status, self-rated health, insurance, self-history of CVD, family CVD history, religiosity, social support, education, income, employment, anger, depression, chronic stress, discrimination, neighborhood deprivation, neighborhood safety, and all possible product terms between optimism, visit, and psychosocial risk.

^†^ P-values were obtained from a global chi-squared test to examine whether at least one of the product term coefficients between optimism and psychosocial risk was different from zero.

**Supplemental Table 2.** Assessment of effect measure modification of adjusted risk ratios* (aRR) for the relationship between optimism at Exam 2 in MESA and the Second Annual Follow-up Interview in JHS and lower cardiovascular risk (0-1 poor metrics) compared with non-lower cardiovascular risk (2-4 poor metrics) using the four biological Life’s Simple 7 metrics (BMI, blood pressure, cholesterol, and glucose) by visit and levels of psychosocial risk measures among MESA and JHS participants included in the secondary analysis sample (N =5,541).

| **Psychosocial risk measure**  **(Potential effect measure modifier)** | **High versus low optimism:**  **aRR for lower CV risk (0-1 poor metrics) by visit and psychosocial risk levels** | | | | | | **Medium versus low optimism:**  **aRR for lower CV risk (0-1 poor metrics) by visit and psychosocial risk levels** | | | | | | **p**^†^ |
| --- | --- | --- | --- | --- | --- | --- | --- | --- | --- | --- | --- | --- | --- |
|  | **Visit 1** | | **Visit 2** | | | | **Visit 1** | | | **Visit 2** | | |  |
|  | **aRR** | **95% CI** | | **aRR** | | **95% CI** | **aRR** | **95% CI** | **aRR** | | **95% CI** |  | |
| **Education at Exam 1** |  |  | |  | |  |  |  |  | |  |  | |
| College degree or more | 1.03 | (0.99-1.08) | | 0.97 | | (0.92-1.02) | 1.01 | (0.97-1.06) | 1.03 | | (0.98-1.07) | 0.89 | |
| High school or some college | 1.01 | (0.97-1.06) | | 1.00 | | (0.95-1.06) | 0.99 | (0.94-1.03) | 1.01 | | (0.96-1.07) |  |  |
| Less than high school | 1.03 | (0.95-1.12) | | 0.95 | | (0.84-1.08) | 1.03 | (0.94-1.11) | 0.98 | | (0.86-1.11) |  |  |
| **Employment at Exam 1** |  |  | |  | |  |  |  |  | |  |  | |
| Employed | 1.02 | (0.99-1.06) | | 1.01 | | (0.96-1.06) | 1.02 | (0.98-1.06) | 1.03 | | (0.99-1.08) | 0.41 | |
| Unemployed | 1.02 | (0.98-1.06) | | 0.95 | | (0.90-1.01) | 0.98 | (0.94-1.03) | 1.00 | | (0.95-1.05) |  |  |
| **Income at Exam 1** |  |  | |  | |  |  |  |  | |  |  | |
| $50,000+ | 1.04 | (1.01-1.09) | | 1.02 | | (0.97-1.07) | 1.03 | (0.99-1.07) | 1.06 | | (1.01-1.11) | 0.50 | |
| $20,000-$49,999 | 1.00 | (0.96-1.05) | | 0.94 | | (0.87-1.01) | 0.98 | (0.93-1.02) | 0.99 | | (0.93-1.05) |  |  |
| $0-$19,999 | 1.00 | (0.94-1.07) | | 0.99 | | (0.91-1.08) | 1.00 | (0.94-1.07) | 0.98 | | (0.89-1.08) |  |  |
| **Anger at Exam 1** |  |  | |  | |  |  |  |  | |  |  | |
| Low | 1.01 | (0.96-1.05) | | 1.01 | | (0.95-1.08) | 1.02 | (0.97-1.07) | 1.05 | | (0.99-1.12) | 0.07 | |
| Medium | 0.99 | (0.95-1.04) | | 1.00 | | (0.93-1.06) | 0.96 | (0.92-1.01) | 1.06 | | (1.01-1.12) |  |  |
| High | 1.09 | (1.03-1.15) | | 0.94 | | (0.87-1.01) | 1.02 | (0.96-1.08) | 0.94 | | (0.88-1.00) |  |  |
| **Depression at Exam 1** |  |  | |  | |  |  |  |  | |  |  | |
| Low | 1.02 | (0.99-1.05) | | 0.99 | | (0.95-1.03) | 1.00 | (0.97-1.03) | 1.03 | | (0.99-1.07) | 0.41 | |
| High | 1.08 | (0.99-1.19) | | 0.97 | | (0.86-1.10) | 1.03 | (0.95-1.11) | 0.93 | | (0.83-1.05) |  |  |
| **Chronic stress at Exam 1** |  |  | |  | |  |  |  |  | |  |  | |
| Low | 1.01 | (0.97-1.05) | | 1.00 | | (0.95-1.05) | 0.99 | (0.96-1.03) | 1.03 | | (0.99-1.07) | 0.34 | |
| Medium | 1.06 | (1.01-1.12) | | 0.96 | | (0.90-1.03) | 1.04 | (0.98-1.10) | 1.03 | | (0.97-1.09) |  |  |
| High | 0.99 | (0.92-1.07) | | 0.99 | | (0.89-1.09) | 0.96 | (0.90-1.03) | 0.98 | | (0.90-1.07) |  |  |
| **Discrimination at Exam 1** |  |  | |  | |  |  |  |  | |  |  | |
| Low | 1.00 | (0.96-1.04) | | 0.99 | | (0.93-1.05) | 1.00 | (0.95-1.05) | 1.06 | | (1.01-1.13) | 0.02 | |
| Medium | 1.02 | (0.97-1.07) | | 0.96 | | (0.90-1.02) | 1.04 | (1.00-1.09) | 0.96 | | (0.90-1.02) |  |  |
| High | 1.07 | (1.01-1.13) | | 1.01 | | (0.94-1.09) | 0.96 | (0.90-1.01) | 1.04 | | (0.97-1.11) |  |  |
| **Neighborhood deprivation at Exam 1** | | | | |  |  |  |  |  | |  |  | |
| Low | 1.02 | (0.98-1.06) | | 0.99 | | (0.95-1.04) | 1.02 | (0.99-1.06) | 1.01 | | (0.97-1.06) | 0.62 | |
| Medium | 1.02 | (0.97-1.08) | | 1.00 | | (0.94-1.07) | 0.98 | (0.93-1.03) | 1.03 | | (0.97-1.08) |  |  |
| High | 1.03 | (0.97-1.09) | | 0.95 | | (0.87-1.04) | 1.00 | (0.93-1.07) | 1.01 | | (0.93-1.11) |  |  |
| **Neighborhood safety at Exam 1** | |  | |  | |  |  |  |  | |  |  | |
| Safe | 1.02 | (0.99-1.05) | | 0.98 | | (0.94-1.02) | 1.01 | (0.98-1.04) | 1.02 | | (0.98-1.06) | 0.23 | |
| Not safe | 1.05 | (0.98-1.12) | | 1.01 | | (0.91-1.12) | 0.97 | (0.90-1.05) | 1.01 | | (0.91-1.11) |  |  |

Note: Clustering of observations by neighborhood, was used in each outcome model. Clustering by neighborhood should also account for within subject correlation in the outcome when subjects are nested within neighborhoods (76).Clustering by subject did not change inference.

^*^ Adjusted for visit, age, gender, race, nativity, geographic region, marital status, self-rated health, insurance, self-history of CVD, family CVD history, religiosity, social support, education, income, employment, anger, depression, chronic stress, discrimination, neighborhood deprivation, neighborhood safety, and all possible product terms between optimism, visit, and psychosocial risk.

^†^ P-values were obtained from a global chi-squared test to examine whether at least one of the product term coefficients between optimism and psychosocial risk was different from zero.
