# Supplementary figures and images for: Examining Optimism, Psychosocial Risks, and Cardiovascular Health Using Life's Simple 7 Metrics in the Multi-Ethnic Study of Atherosclerosis and the Jackson Heart Study
Source: Front Cardiovasc Med. 2021 Dec 15;8:788194. doi: 10.3389/fcvm.2021.788194 (PMC8714850; doi:10.3389/fcvm.2021.788194)

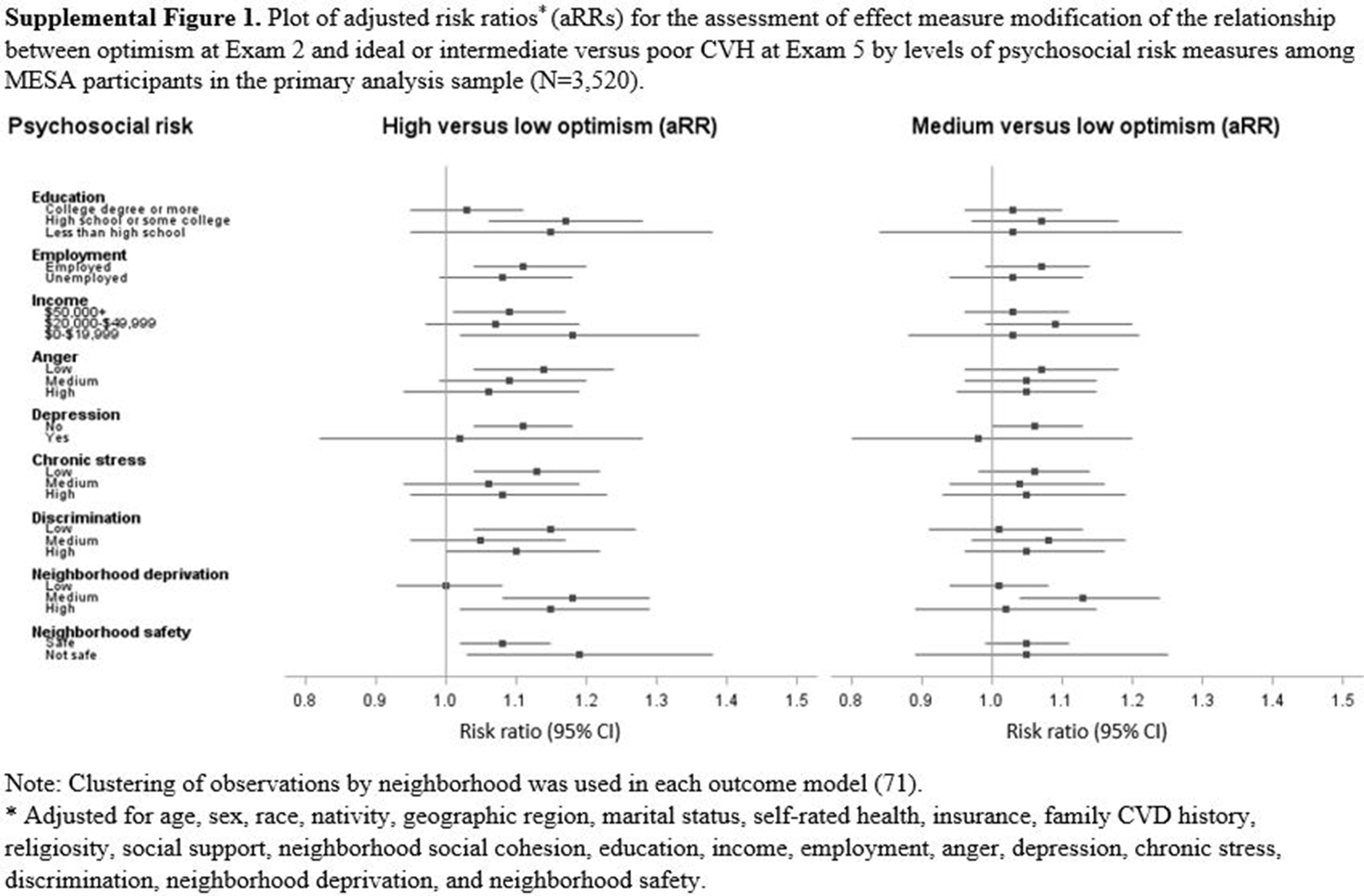

Supplement: Supplementary file 2 [file Image_1.jpg]

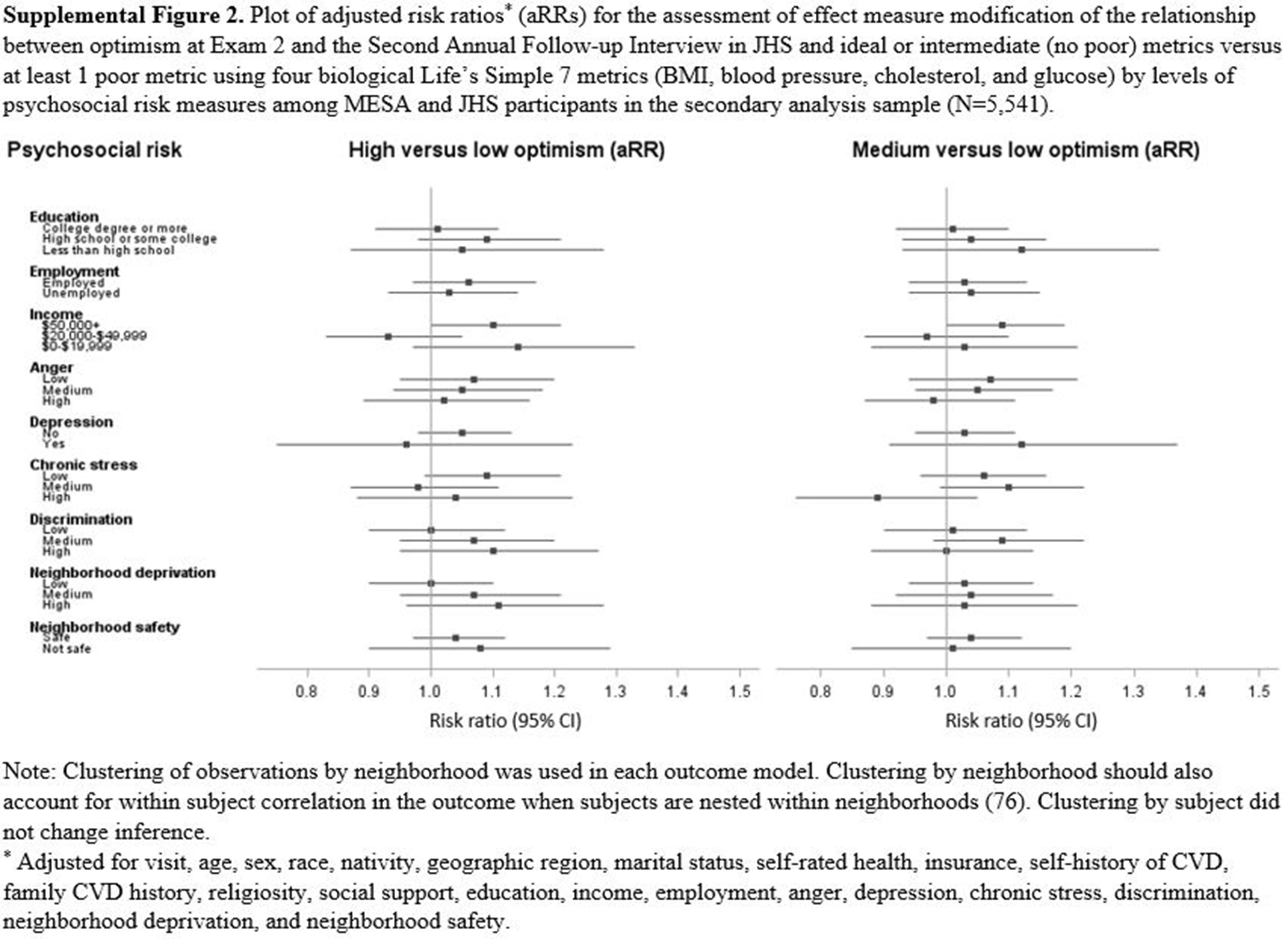

Supplement: Supplementary file 3 [file Image_2.jpg]

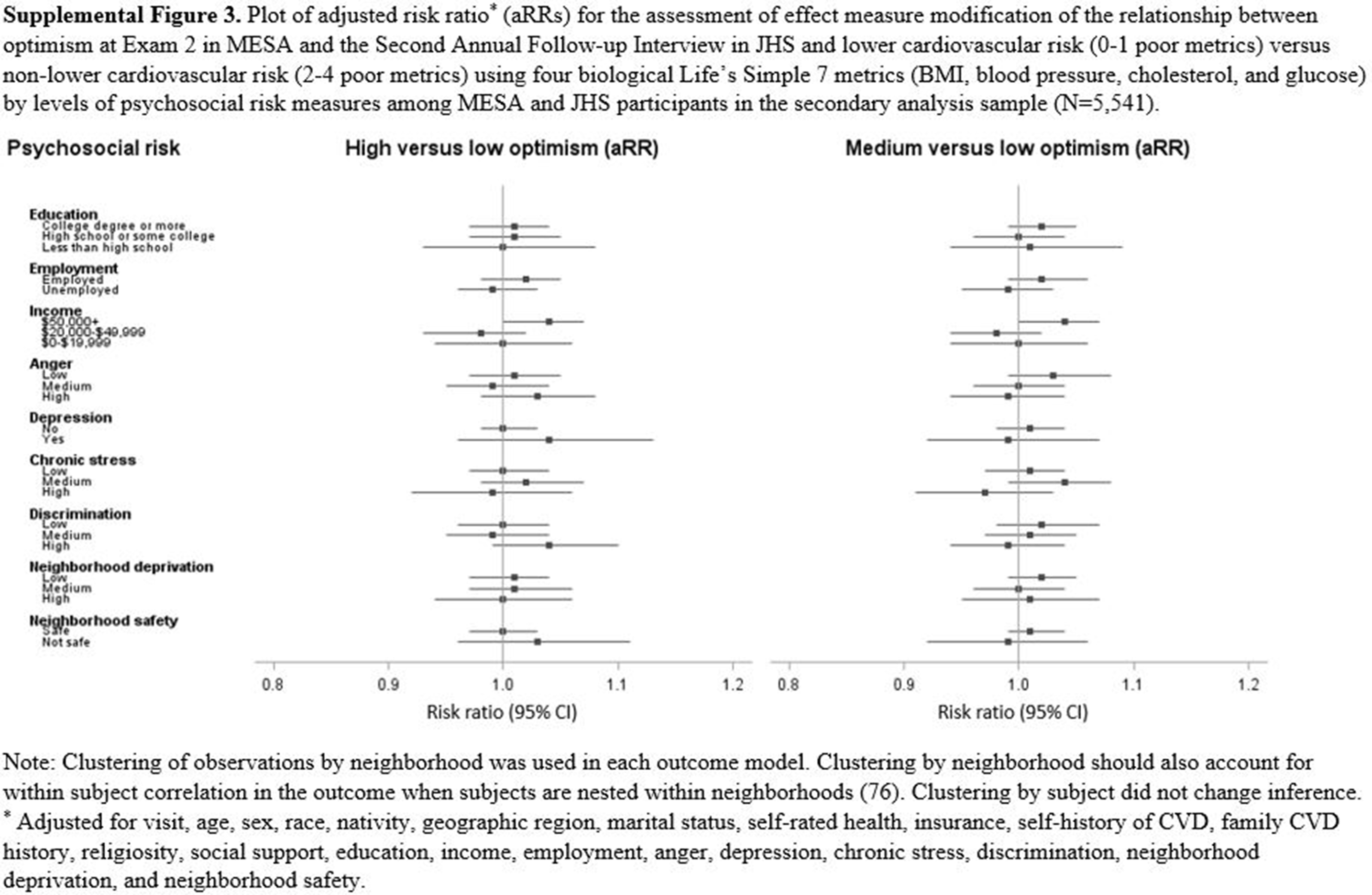

Supplement: Supplementary file 4 [file Image_3.jpg]
